# Accuracy and Workflow Improvements for Responsive Neurostimulation Hippocampal Depth Electrode Placement Using Robotic Stereotaxy

**DOI:** 10.3389/fneur.2020.590825

**Published:** 2020-12-10

**Authors:** Patrick J. Karas, Nisha Giridharan, Jeffrey M. Treiber, Marc A. Prablek, A. Basit Khan, Ben Shofty, Vaishnav Krishnan, Jennifer Chu, Paul C. Van Ness, Atul Maheshwari, Zulfi Haneef, Jay R. Gavvala, Sameer A. Sheth

**Affiliations:** ^1^Department of Neurosurgery, Baylor College of Medicine, Houston, TX, United States; ^2^Department of Neurology, Comprehensive Epilepsy Center, Baylor College of Medicine, Houston, TX, United States

**Keywords:** hippocampal depth electrode, robotic stereotaxy, responsive neurostimulation (RNS), RNS workflow, robotic stereotaxy accuracy, NeuroPace

## Abstract

**Background:** Robotic stereotaxy is increasingly common in epilepsy surgery for the implantation of stereo-electroencephalography (sEEG) electrodes for intracranial seizure monitoring. The use of robots is also gaining popularity for permanent stereotactic lead implantation applications such as in deep brain stimulation and responsive neurostimulation (RNS) procedures.

**Objective:** We describe the evolution of our robotic stereotactic implantation technique for placement of occipital-approach hippocampal RNS depth leads.

**Methods:** We performed a retrospective review of 10 consecutive patients who underwent robotic RNS hippocampal depth electrode implantation. Accuracy of depth lead implantation was measured by registering intraoperative post-implantation fluoroscopic CT images and post-operative CT scans with the stereotactic plan to measure implantation accuracy. Seizure data were also collected from the RNS devices and analyzed to obtain initial seizure control outcome estimates.

**Results:** Ten patients underwent occipital-approach hippocampal RNS depth electrode placement for medically refractory epilepsy. A total of 18 depth electrodes were included in the analysis. Six patients (10 electrodes) were implanted in the supine position, with mean target radial error of 1.9 ± 0.9 mm (mean ± SD). Four patients (8 electrodes) were implanted in the prone position, with mean radial error of 0.8 ± 0.3 mm. The radial error was significantly smaller when electrodes were implanted in the prone position compared to the supine position (*p* = 0.002). Early results (median follow-up time 7.4 months) demonstrate mean seizure frequency reduction of 26% (*n* = 8), with 37.5% achieving ≥50% reduction in seizure frequency as measured by RNS long episode counts.

**Conclusion:** Prone positioning for robotic implantation of occipital-approach hippocampal RNS depth electrodes led to lower radial target error compared to supine positioning. The robotic platform offers a number of workflow advantages over traditional frame-based approaches, including parallel rather than serial operation in a bilateral case, decreased concern regarding human error in setting frame coordinates, and surgeon comfort.

## Introduction

Robotic stereotaxy is increasingly common both for the implantation of stereo-electroencephalography (sEEG) electrodes during phase 2 epilepsy monitoring and for the implantation of permanent therapeutic neuromodulation systems such as deep brain stimulation (DBS) and responsive neurostimulation (RNS) (1). Similar to frame-based stereotactic systems, robotic stereotaxy allows for submillimeter accuracy for the implantation of DBS electrodes (2, 3), which has broadened its application across the United States and Europe. Advances in computer planning software have also facilitated broader adoption.

In this paper we discuss our use of robotic stereotaxy for the placement of occipital-approach hippocampal depth electrodes for RNS therapy. We describe the evolution of our workflow, which started with the supine beach-chair semi-sitting positioning for implantation of depth electrodes and neurostimulator in one stage with evolution to prone implantation of hippocampal depth electrodes followed by supine implantation of the RNS neurostimulator in two stages. We present a technical note on our electrode implantation workflow, and our initial single center experience with electrode implantation accuracy and early seizure response rates.

## Materials and Methods

### Subjects

**Figure 1:**
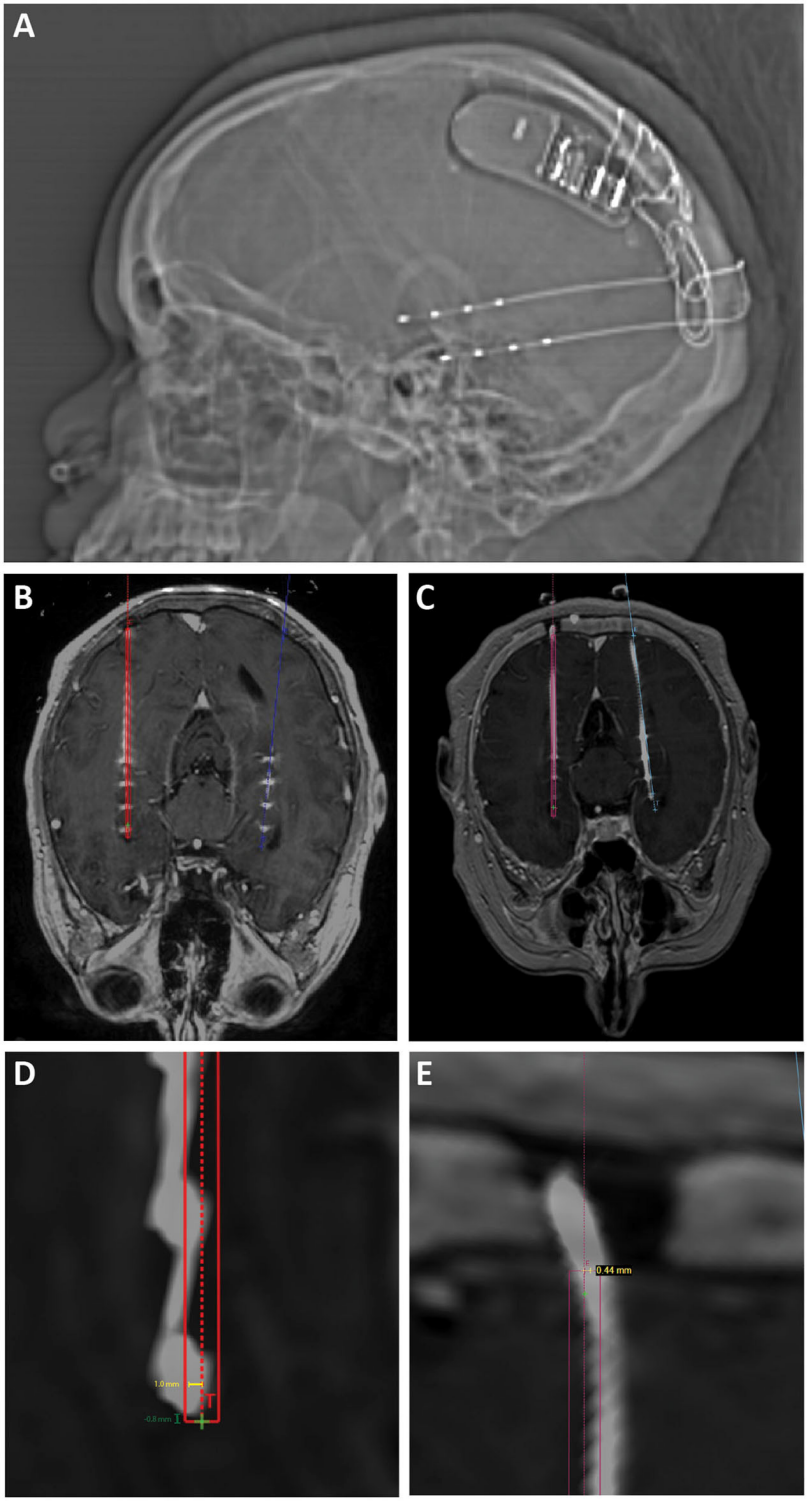
**(A)** Scout x-ray showing lateral view after implantation of bilateral hippocampal RNS electrodes and RNS generator. **(B)** Intra-operative O-arm fluoroscopic CT projected over preoperative planning MRI is used to confirm target accuracy electrodes compared to operative plans (displayed in red and blue). **(C)** Post-operative CT projected over preoperative planning MRI was also used to confirm target accuracy in cases where O-arm fluoroscopic CT was not performed. **(D)** Radial target error (yellow line) was measured as the distance from the planned electrode target to the center of the actual electrode position. Depth target error (green) was measured as the difference in depth between the implanted electrode and the planned electrode tip measured along the trajectory of the implanted electrode. Positive values represent electrodes that were implanted past/deeper to target. Negative values represent electrodes that were implanted more shallow compared to target. **(E)** Radial entry point error was measured as the distance from the planned electrode entry point at the inner table of the skull to the center of the implanted electrode.

Ten consecutive subjects (6 women and 4 men) who underwent unilateral or bilateral hippocampal RNS depth electrode implantation (Figure 1A) were included. The median age was 40 years (range 25–63), with median duration of epilepsy for 15 years (range 3–43 years). The subjects failed a median of 4 antiepileptic medications prior to RNS implantation (range 2–12 medications) and reported a median seizure frequency of 1–2 seizures weekly. Full demographic information is available in Table 1.

**Table 1:** Patient demographics.

**Patient number**	**Age**	**Duration of epilepsy (years)**	**Failed AEDs**	**Seizure frequency**	**Seizure onset zone**	**MRI findings**	**Aura**	**Semiology**	**Prior Surgery**	**RNS Implant**
1	45–49	31	11	1–2/week	Left mesial temporal	L MTS	Fear, warmth	Staring, automatisms	Right ATL; VNS (removed)	L hippocampus; L temporal strip
2	60–64	43	4	1–3/week	Bilateral mesial temporal	Bilateral MTS	None	Slowed blinking; LOC; generalized convulsions	none	bilateral hippocampal
3	40–44	4	3	1–2/month	Left inferior temporal gyrus	L incomplete hippocampal inversion	Weird feeling	Alexia, aphasia, impaired awareness; GTC	none	L hippocampus; L anterior subtemporal strip
4	25–29	16	3	10–12/day	Left temporal	L MTS; tectal glioma	None	Left eye gaze, hand dystonia, amnesia, motor aphasia, oral automatisms	numerous surgeries for tumor and VP shunt	L hippocampus; L parahippocampus
5	30–34	4	4	1/week	Left mesial temporal	None	None	Loss of awareness, behavioral arrest	none	bilateral hippocampal
8	25–29	3	2	2/week	Bilateral mesial temporal	Possible small L temporal encephalocele	None	Growling noise; eyes roll back; body stiffening and shaking	none	bilateral hippocampal
6	60–64	23	12	1/week	Bilateral mesial temporal	None	Rotten meat smell	Salivation, behavioral arrest, lip smacking, confusion	none	bilateral hippocampal
7	55–59	37	5	1–2/week	Left mesial temporal	L MTS	Chills	Staring, right hand posturing, motor automatisms, head turn, LOC	none	bilateral hippocampal; L inferior temporal lobe strip
9	35–39	9	4	<1/month	Bilateral mesial temporal	L MTS	Bilateral arm tingling	Loss of awareness, vocalization, body shaking	none	bilateral hippocampal; L subtemporal strip
10	35–39	14	7	1–2/week	Bilateral mesial temporal	None	déjà vu	Staring, perseveration, bitter taste, altered awareness, orolingual automatisms	none	bilateral hippocampal

*All patients had medically refractory epilepsy with seizure onset zone either in the left mesial temporal or bilateral mesial temporal regions. These patients were not considered to be candidates for resective or ablative surgeries after review at a multidisciplinary epilepsy conference. AED, antiepileptic drug; ATL, anterior temporal lobectomy; GTC, generalized tonic clonic; L, left; LOC, loss of consciousness; MTS, mesial temporal sclerosis; VNS, vagus nerve stimulator*.

### Procedure

Preoperative high-resolution CT and MRI scans are loaded into the ROSA (Zimmer Biomet, Warsaw, Indiana, USA) planning computer and merged. A preoperative CT is highly recommended, as intraoperative fluoroscopic CT imaging (O-arm O2, Medtronic, Minneapolis, MN, USA) merges best with CT; merging these images to MRI may result in suboptimal registration. The ROSA software automatic registration is used but must be checked carefully prior to proceeding with planning and implantation. The target trajectory length is set to 190 mm (based on cannula length; see below), and the intended electrode trajectory is planned based on the surgeon's standard procedure. We use an occipital approach and strive for a trajectory that spans the head and body of the hippocampus, often extending into the amygdala, with >25 mm of the trajectory within these structures. We use a T1 post-contrast MRI in order to identify and avoid traversing vascular structures and also try to avoid entering the occipital horn of the lateral ventricle in order to avoid deflection off the ependymal surface.

Prior to using robotic stereotaxy, we used a conventional frame for RNS depth electrode placement. For an occipital approach hippocampal depth electrode, we would position the patient supine in a slouched semi-sitting position with the neck flexed, such that the trajectory is approximately parallel to the floor. This position is not easily achieved with our preferred head fixation method using the robot (see below) because of the low occipital entry points and inferior-to-superior trajectory. We therefore evolved our approach to performing the electrode implant procedure prone, with a second stage for placement of the RNS generator. In some cases, the two stages were performed during the same procedure, but in others we separated them into separate admissions as is common in DBS surgery. The prone position during stage 1 facilitates placement of the hippocampal depth electrodes. The second stage, generator placement and connection to the depth leads, is performed supine with the head turned. This is the approach we describe in this report.

**Figure 2:**
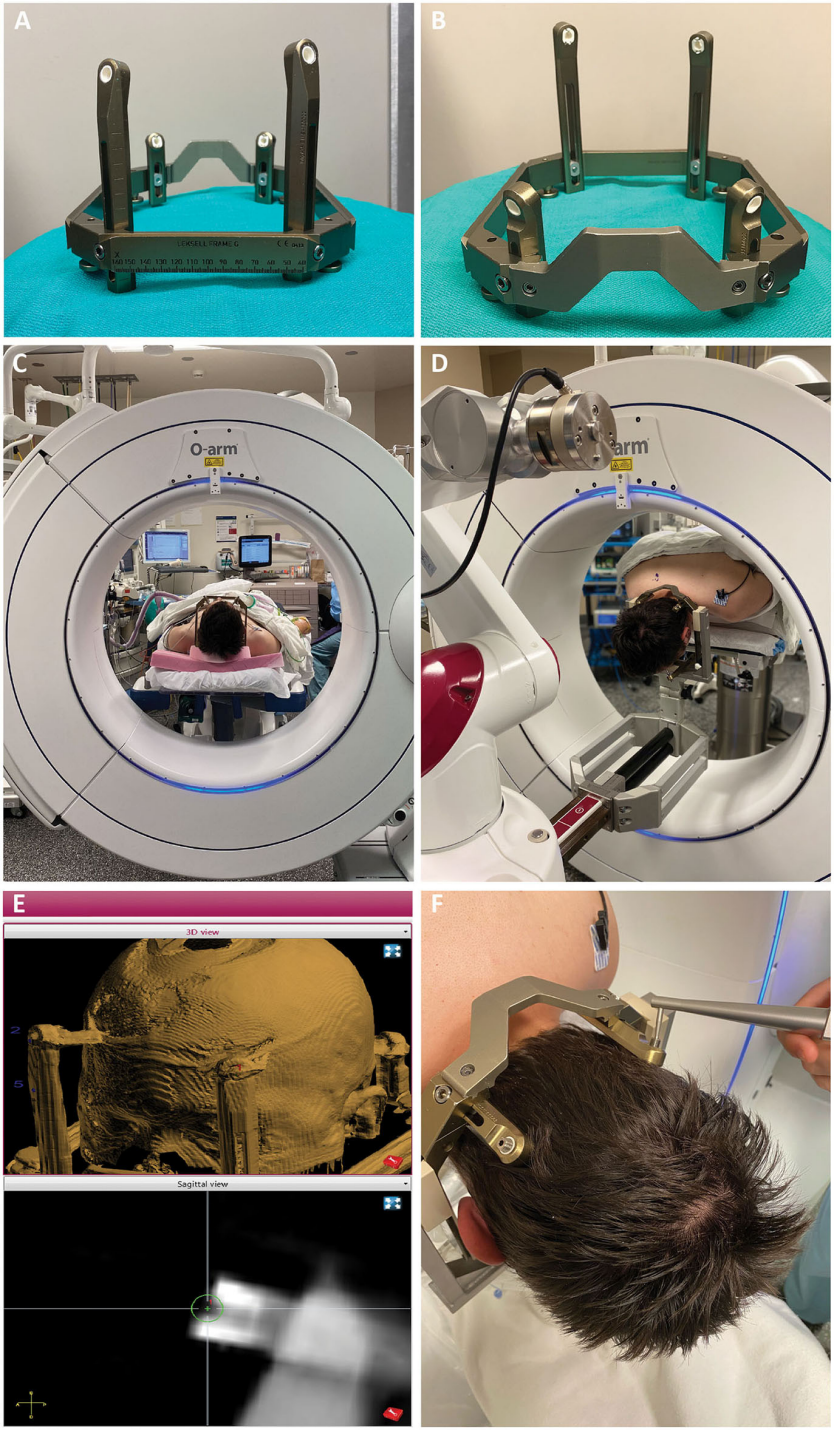
**(A,B)** The Leksell frame is assembled opposite the traditional manner, with the short fixation posts flanking the curved nasal piece, and long fixation posts flanking the straight piece. Female fixation screws are used and serve as skull fiducials for robot registration after O-arm imaging. **(C)** After frame placement, the patient is initially positioned supine while still on the stretcher, with the head supported on a radiolucent plastic board, and a pre-operative O-arm image is obtained for registration to the pre-operative CT. **(D)** The patient is then flipped prone on gel rolls on the OR table, and the Leksell frame is affixed to the robot using the goalpost-shaped Leksell holder. The reverse orientation of the frame allows the three straight edges of the frame to fit within the beveled clamps of the Leksell holder, with the curved nasal piece over the occipital region. **(E)** Registration points are chosen on the merged fluoroscopic CT image including the frame. Four points are chosen, one for each frame pin, such that the registration marker sits in middle of the divot on the pin with its equator flush with the flat surface of the pin. **(F)** Registration is then performed using the ball-tip probe robot attachment.

The patient is induced under general endotracheal anesthesia on the transport stretcher. The back of the stretcher can be easily raised to facilitate placement of the Leksell frame, which we use for both head fixation and robot registration. The Leksell frame is assembled “backwards” so that the short fixation posts are alongside the curved nasal front piece and the long fixation posts are along the straight portion of the frame (Figures 2A,B). The frame is still applied with the short posts on the occipital aspect and the front posts on the frontal aspect of the head, but the reverse assembly of the frame puts the curved nasal piece over the occipital aspect.

There are two types of Leksell frame pins available. We use the pins with a female head, as this socket allows for robot registration. The frame pins thus serve as skull fiducials, allowing the accuracy of skull fiducial-based registration without the need for placement of separate fiducials screws. The long fixation posts are placed at slightly different lengths creating non-coplanar registration points (Figures 2A,B). Non-coplanar registration points are important to ensure that the registration algorithm arrives at a unique (i.e., correct) solution. After applying the frame, we place a radiolucent board under the stretcher mattress and slide the patient up so the head is on the board. The fluoroscopic CT system (O-arm O2, Medtronic, Minneapolis, MN, USA) is then positioned around the head, and we obtain the fluoroscopic CT including the frame in the field of view (Figure 2C). This process allows the acquisition of a suitable scan in the supine position, before flipping prone, and without metallic artifact. This fluoroscopic CT is then merged with the preoperative planning CT (1 mm axial cuts) on the ROSA robot.

The patient is then turned prone onto the OR table on gel rolls, and the frame is affixed to the “goalpost-shaped” ROSA Leksell holder (Figure 2D). This Leksell holder has fewer degrees of freedom than the Mayfield-style Leksell holder, making supine placement of occipital approach hippocampal depths difficult, but is extremely stable and sturdy. Once the frame is affixed, the OR table and robot are locked and should not be moved, as the head is affixed to the robot, and the body to the table. Ensuring that the bed control is off and the bed unplugged has been incorporated into our Time Out procedure.

Registration begins with choosing the registration points on the fluoroscopic CT (Figure 2E). The 3 mm diameter registration circle fits snugly within the divot of the frame pin, with little room for radial ambiguity. The depth ambiguity is reduced by choosing a depth that puts the equator of the registration circle at the flush surface of the frame pin. The surgeon then navigates the ball-tip registration attachment to each point and marks it (Figure 2F). Again, there is little room for laxity in the radial position for choosing this point given the close fit of the ball tip within the frame pin, and the depth position is chosen by placing the equator of the ball tip at the level of the surface of the pin. We strive for a registration error (root mean square, RMS) of <0.8 mm and are usually able to achieve <0.6 mm. After the subsequent verification step, we navigate to and mark the entry point(s). The fluoroscopic CT is then positioned over the patient to be draped in for intraoperative verification during the procedure. We plan for a ~1 inch incision at each entry point and prep and drape these regions.

**Figure 3:**
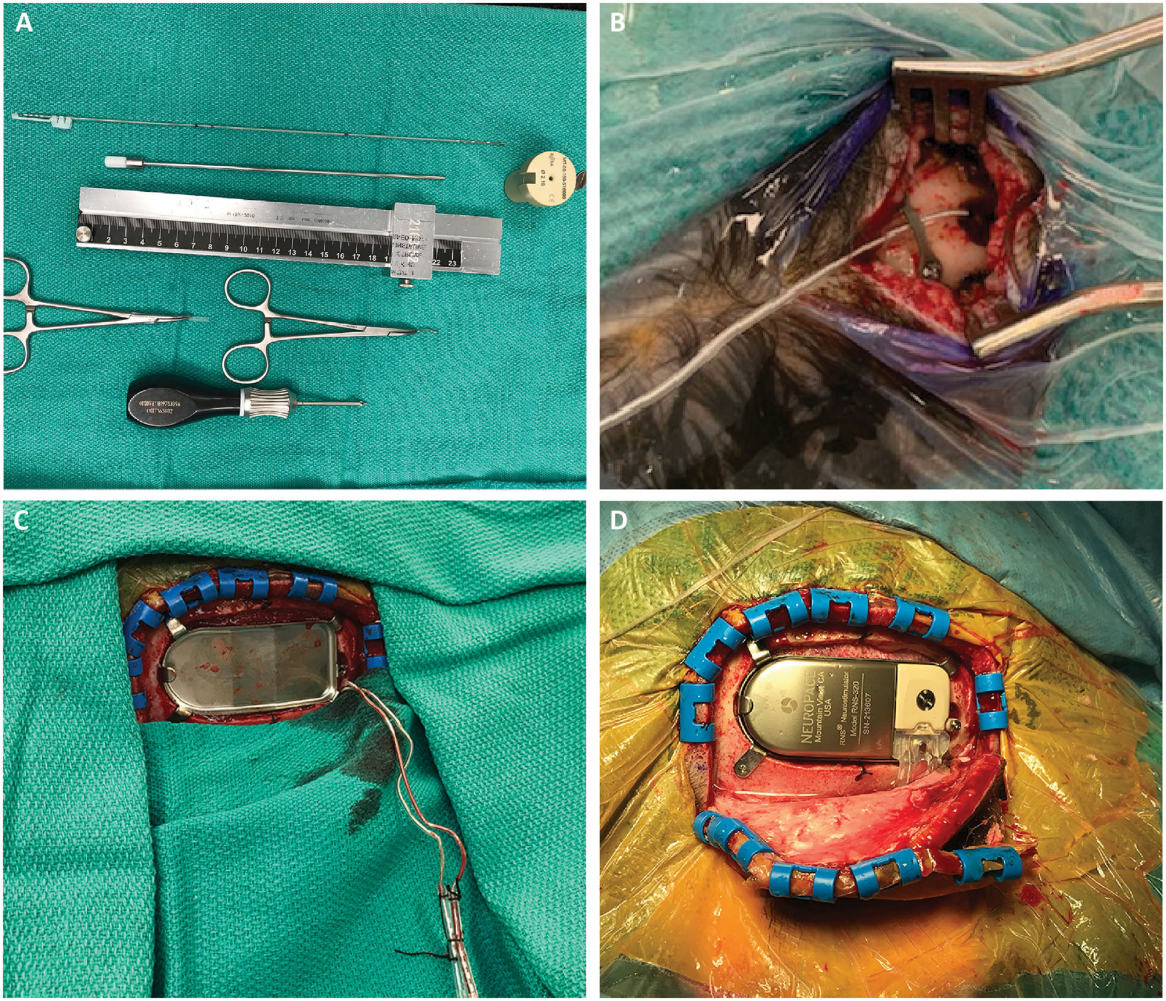
**(A)** Required stereotactic equipment for the procedure: 2.15 mm PEEK ROSA adapter, 190 mm length slotted canula, RNS depth electrode, stereotactic ruler, bent cranial plate and cut segment of lead cap. The depth electrode is marked at three points: 190 and 200 mm, which flank the wide hub of the cannula, and the point corresponding to the outer table of the skull, measured on the stereotactic plan. This last point is important to mark, as it is the only one visible once the cannula is removed and the robot arm moved away. **(B)** After the electrode is inserted, a dog-bone cranial fixation miniplate is fastened to the skull to hold the electrode in place. We use the cut segment of the lead cap as a shock absorber around the lead. **(C)** After creating the craniectomy and securing the ferrule, the RNS leads are retrieved from their subgaleal position. **(D)** The RNS generator can be placed and connected to the depth electrodes. This can be performed either in the same prone position or after re-positioning the patient supine with the head turned.

We create vertical linear incisions over the entry points and use a cutting burr to create small burr holes at each entry point. The burr hole can also be created with a twist-drill bit through the robot arm. If doing so, we recommend over-sizing the hole (e.g., with a 3.2 mm bit) in order to ensure that the cannula does not deflect off the bone edge. We use the 2.15 mm PEEK ROSA adapter attachment for the robot, which fits the slotted cannula (2.1 mm, Ad-Tech Medical Instrument Corporation, Oak Creek, WI, USA) that we use for electrode placement (Figure 3A). This slotted cannula is 190 mm in length, which is why each trajectory is defined as 190 mm long. In the “Axial Fast” mode within each trajectory, the robot arm and cannula can be quickly brought in and out to ensure that the burr hole is centered around the cannula as it is drilled. Because there are no frame coordinates to set and check between trajectories, a surgeon and assistant can work in parallel by handing the robot arm back and forth, thus greatly increasing efficiency in a bilateral case.

We pass monopolar cautery (set at 20) through the cannula to open the dura just around the cannula, thus minimizing CSF loss. With the robot arm at target, the slotted canula is advanced to full depth, placing it at target, and the inner stylet removed. Prior to placing the RNS (10 mm inter-contact spacing, NeuroPace Inc., Mountain View, CA, USA) depth lead, we mark it at three points: 190 and 200 mm, which flank the wide hub of the cannula, and the point corresponding to the outer table of the skull (measured on the robot, Figure 3A). Following placement of the lead into the cannula, the lead is bent out of the slot in the cannula, and the cannula is removed. The lead is held steady at the skull, visualizing the bottom pre-placed mark at the outer table. The lead stylet is then removed, and the robot arm is moved out of the way. At this point the only visual indication of depth is the mark at the outer table. The electrode is folded over and affixed to the skull with a “dog-bone” cranial plate, with a short cylinder of lead cap used as a shock absorber around the lead (Figure 3B). If using a smaller twist-drill burr hole, we recommend beveling the edge of the hole to prevent the sharp edge from damaging the lead over time. After placement of both leads, we obtain an intraoperative fluoroscopic CT, register it to the preoperative CT on the stereotactic plan, and verify lead location relative to the planned trajectory (Figure 1B). If accuracy is acceptable, we place temporary caps on the leads, mark them with our conventional marks to maintain laterality, and tunnel them subgaleally to the planned location of the RNS generator. The incisions are then irrigated and closed.

The stage 2 surgery is performed supine with the head turned. The RNS generator is placed using standard technique, typically in the right parietal area. Once the craniectomy is performed and the ferrule is secured, the previously tunneled leads are exposed and connected to the generator (Figures 3C,D). In some cases in our series, this stage 2 surgery followed immediately after the stage 1 surgery after re-positioning and re-prep/draping. In other cases, we separated the two stages with a 1 week interval, allowing the patient to go home between the stages, similar to the approach for DBS. In either case, we obtain a stereotactic head CT after stage 1 (if staged) or the whole procedure (if combined).

For comparison, we also briefly mention the supine method of placement used in our earlier cases. Notable differences are placing the Leksell frame in the usual front-facing orientation and using the Mayfield-style robot attachment to attach the frame to the robot. This arrangement allows better elevation and flexion of the head in order to produce an achievable angle for the occipital entry, but we found the operating position uncomfortable for drilling the burr hole and visualizing the cannula entry point on the dura. For these reasons we adopted the prone approach described above.

### Analysis

Patient charts were retrospectively reviewed with institutional review board approval. Registration was performed using the automatic registration tool in the ROSA planning software and confirmed with manual inspection. All scans, including pre-operative planning MRI, intraoperative O-arm spins (Figure 1B), and post-operative CT scans (Figure 1C), were registered to a stereotactic pre-operative CT scan.

Radial target error (Figure 1D, yellow measurement) was measured as the distance from the planned electrode target to the center of the actual electrode position in the “Trajectory View” at the deepest point of the planned electrode trajectory. This measurement was confirmed on a “Cross-Sectional” view. Depth target error (Figure 1D, green measurement) was measured as the difference in depth between the actual electrode and the planned electrode tip, either deeper (positive) or more shallow (negative). Radial entry point error (Figure 1E) was measured as the distance from the planned electrode entry point at the inner table of the skull to the center of the actual electrode in the “Trajectory View.” The trajectory length was measured as the distance from the inner table of the skull to the tip of the planned electrode position along the planned trajectory of the electrode.

Seizure outcomes were reported using proxy values obtained from the RNS recordings. We reviewed ECoG recordings and stimulations during the first month after the initial post-implantation detection stabilization. “Long events” identified by the RNS device were used as a proxy for seizure events, as home seizure diaries were not available for some of the patients implanted. All long events identified by the RNS were manually examined and only retained if they exhibited sustained rhythmic activity with epileptiform evolution. Daily activations were also recorded and averaged over the same month to calculate average daily activations. The same process was followed at 6 months after post-implantation detection stabilization and during the last month of available data (last follow-up). Responder rate was defined as the percentage of subjects with a 50% or greater reduction in seizure frequency.

All calculations were performed in Microsoft Excel. Reported errors were calculated using standard deviation. One-tailed unpaired *t*-test was used to determine statistical significance for comparisons of radial target error, radial entry point error, and CT vs. O-arm measurements. Two-tail unpaired *t*-test was used to determine statistical significance for the comparison of depth target error. We set statistical significance at *p* < 0.01 after Bonferroni correction for multiple comparisons (*p* < 0.05/4; *m* = 4). Linear correlation between trajectory length and radial target error was calculated using the Pearson correlation coefficient.

## Results

Ten consecutive patients who underwent robotic RNS hippocampal depth electrode implantation over 16 months were retrospectively included in this study. All ten patients' original implantation plans were available for review, totaling 18 depth electrodes. The locations of 8 electrodes (five patients) were measured with post-operative CT. The locations of 10 electrodes (five patients) were measured with intra-operative O-arm. Eight electrodes across four patients were implanted in patients positioned prone, and ten electrodes across six patients were implanted in supine procedures.

### Electrode Placement

Electrode placement results are compiled in Table 2. The mean radial error across all patients was 1.4 ± 0.9 mm. Mean radial target error for the prone position was 0.8 ± 0.3 mm, significantly less than the 1.9 ± 0.9 mean radial target error in the supine position (*p* = 0.002). The mean depth error across all patients was past target by 2.9 ± 3.6 mm. There was no significant difference between the prone position (deep by 2.7 ± 2.3 mm) and the supine position (deep by 3.0 ± 3.8 mm, *p* = 0.31). The mean entry point radial error across all patients was 0.9 ± 0.7 mm with no significant difference between the prone position (0.8 ± 0.7 mm) and the supine position (0.9 ± 0.7 mm, *p* = 0.39). There was not a strong correlation between trajectory length and radial target error across all electrodes (*r* = 0.26). There was no significant difference between radial target error measured by intraoperative O-arm (1.2 ± 0.9 mm) vs. post-operative CT (1.6 ± 0.8 mm, *p* = 0.14).

**Table 2:** Electrode measurements across all patients.

**Patient number**	**Electrode number**	**Electrode location**	**Position**	**Electrode location confirmation**	**Radial target error**	**Depth error**	**Entry point radial error**	**Trajectory length**
1	1	L HC	Supine	CT	2.7	0.0	0.5	92.6
2	1	R HC	Supine	CT	1.8	3.7	1.8	86.0
	2	L HC			2.4	3.3	0.4	88.9
3	1	L HC	Supine	CT	0.8	4.4	0.5	102.9
4	1	L HC	Supine	O-arm	3.0	5.8	0.1	97.4
	2	L para HC			2.7	−1.7	0.0	93.0
5	1	R HC	Supine	CT	0.4	−0.9	1.0	102.2
	2	L HC			2.1	−6.3	1.1	103.5
8	1	R HC	Supine	CT	1.0	−0.8	1.9	98.5
	2	L HC			2.0	−3.2	1.8	99.1
6	1	R HC	Prone	O-arm	0.7	−2	0.5	83.3
	2	L HC			0.9	−2.9	2.1	83.5
7	1	R HC	Prone	O-arm	0.3	5.3	0.2	85.7
	2	L HC			0.6	−1.2	0.9	87.7
9	1	R HC	Prone	O-arm	0.6	−1.8	0.5	89.8
	2	L HC			1.1	−7	0.0	92.9
10	1	R HC	Prone	O-arm	1.2	−0.4	0.8	83.1
	2	L HC			0.7	−1	1.5	81.9

*All measurements in mm. Electrodes placed in the prone position had smaller radial target error compared to those placed in the supine position. HC, hippocampus*.

### Seizure Outcomes

Eight of ten patients had enough data after a baseline recording period to report seizure outcome based on RNS recordings and stimulations (Table 3). The two patients who did not have sufficient data recently had their detection parameters changed. Median time from implantation to stabilization of detection was 1.9 months (range 0.6–9.6 months). Median follow-up time after detection stabilization was 7.5 months (range 3.4–10.7 months).

**Table 3:** Seizure outcomes at last follow-up.

**Patient number**	**Monthly long episodes at first month**	**Average daily activations during first month**	**Monthly long episodes at last follow-up**	**% reduction in monthly long episodes at last follow-up**	**Average daily activations at last follow-up**	**% change in daily activations at last follow-up**	**Last follow-up (months)**
1	19	1,300	16	16%	900	31%	10.7
2	32	2,000	41	−28%	2,100	−5%	7.4
3	4	1,250	2	50%	500	60%	7.6
4	43	1,000	52	−21%	1,500	−50%	9.0
5	1	240	0	100%	35	85%	9.4
8	0	800	3	N/A	500	38%	5.7
9	12	150	0	100%	200	−33%	4.6
10	9	1,000	12	−33%	1,250	−25%	3.4

*In our preliminary short-term follow-up (median 7.5 months), mean seizure frequency reduction at last follow-up was 26%. Patients 6 and 7 had insufficient data for seizure outcome collection*.

Mean seizure frequency reduction at last follow-up was 26% (available in eight patients). Five patients (62.5%) had reduction in seizure frequency, and three of these (37.5%) had ≥50% reduction in seizure frequency at last follow-up. Mean reduction in the number of average daily activations at last follow-up was 9%. Four patients (50%) had a reduction in average daily activations at 6 months.

There were no complications, including no cases of intracranial hemorrhage, infection, or hardware failures.

## Discussion

This study describes the evolution and current details of our workflow for robotic stereotactic implantation and intraoperative image-guided verification of occipital approach mesial temporal depth electrode placement for RNS therapy. Robotic stereotaxy assists in accurate placement of hippocampal depth electrodes with submillimeter precision, especially when the patient is positioned prone, according to our data. Intraoperative image-based verification allows the surgeon to confirm that the electrode is in the intended target using volumetric imaging prior to leaving the OR.

Our initial robotic workflow, adopted from our frame-based workflow for both hippocampal RNS leads and for laser amygdalohippocampotomy (4, 5), used the slouched semi-sitting position for occipital-approach mesial temporal trajectories. As mentioned above, the Mayfield attachment of the robot allows this position, but it is fairly uncomfortable for the surgeon and also makes visualizing the full interior of the burr hole more difficult. This circumferential visualization is important for ensuring that the cannula is not deflecting off the edge of the burr hole or the edge of dura. Because the angle of the cannula is horizontal, it may also be more likely to deflect off trajectory due to gravity if there is any play in the adapter holding the cannula. All these factors may introduce errors in stereotactic accuracy. Additional errors in this position can be introduced secondary to cerebrospinal fluid egress since occipital burr holes are in a dependent position.

For all these reasons, we have shifted to the prone position. The setup requires the minor hassle of prone positioning, but the surgeon's comfort, easy view of the burr hole and dural entry point, and vertical trajectory relative to the ground are superior features. As we show here, this position also seems to produce better radial accuracy. Depth accuracy was worse than radial accuracy and did not differ between the positions. We suspect that the greater depth accuracy error is related to the method of securing the lead. Rather than clamping it in place *in situ*, as is typically done during DBS procedures using a lead securing system in the burr hole cover, we visualize a mark and secure the lead to the skull with a cranial miniplate. This method likely leads to slight depth movement of the lead and therefore the larger measured error. Our experience with robotic DBS demonstrates a significantly lower depth error, further implicating the lead securing technique as the cause of the increased error. The RNS depth electrode kit does provide a DBS-style burr hole cover that would allow *in situ* securing, should the surgeon want to try to reduce this depth error. We plan a trajectory spanning 26–30 mm of amygdala and hippocampal tissue, a distance that matches the 32 mm span of contacts across the wide-spaced (10 mm inter-contact distance) depth lead. Thus, a depth error of 2–3 mm is not critical. We feel that the much more important feature is a low radial error, which reduces the chance of being off trajectory enough to hit vascular structures that the trajectory was planned to avoid.

Prior reports of robotic stereotactic implantation for DBS devices have achieved submillimeter accuracy with average radial target errors of 0.86 mm (2, 3), suggesting that for DBS surgery robotic stereotaxy has at least equivalent accuracy compared to frame-based stereotaxy. Direct comparison of robotic *vs*. frame-based stereotaxy has also suggested that robotic stereotaxy has lower mean radial target error of 0.76 ± 0.37 mm compared to 1.11 ± 0.59 mm for frame-based implantation (6). Prior reports for robotic-assisted occipital-approach mesial temporal RNS depth electrode accuracy have not been as positive, with median radial error over 2.18 mm (7). We improved our initial mean radial target error of 1.9 ± 0.9 mm achieved in the supine position with better intraoperative ergonomics and positioning, decreasing it to 0.8 ± 0.3 mm with the prone technique described above.

The incorporation of intraoperative fluoroscopic CT also represents an important improvement in our workflow. It allows us to head fix and register the patient without leaving the OR for a CT scan, reducing procedural time and increasing efficiency. Draping the scanner into the field also allows easy and rapid acquisition of a volumetric image set to verify the location of the lead. Just as image verification prior to leaving the OR is becoming an essential feature of DBS procedures (8–10), we propose the same standard for other stereotactic procedures such as RNS depth electrode placement.

There are several advantages to robotic stereotactic procedures over using a conventional frame. Entry points can be easily marked prior to draping, allowing a smaller hair shave. Although setup may take slightly longer because of the registration process, the time savings during the procedure more than makes up for the setup time cost. The robot arm can move back and forth between the two trajectories of a bilateral implant, allowing the surgeon and assistant to work simultaneously. Resetting the five coordinates for a frame requires serial rather than parallel effort. Not having to set and check these coordinates also reduces the chance for human error and allows trainees to play a greater role, as the surgeon does not have to worry about coordinate errors. On top of these advantages, robotic procedures also do not sacrifice accuracy, as demonstrated here and by several others (2, 3).

One disadvantage to the prone lead placement is the slightly more awkward position for generator placement. It can either be placed in the same procedure, or the patient can be re-positioned supine with the head turned for a more comfortable position. In some cases, we staged the procedures, with prone lead placement in one procedure and supine generator placement and attachment to the leads in a separate procedure 1–2 weeks later. Logistical reasons such as OR time or equipment availability concerns usually drove the choice for staged procedures. The current additional burden of COVID-19 testing required before all elective surgical procedures favors a single-stage procedure.

Reported seizure outcomes are preliminary and include small sample size and relatively short follow-up duration. A 67.9% median reduction in seizure frequency, along with 64.5% responder rate, has been reported in patients with bilateral onset mesial temporal epilepsy observed 2–6 years after bilateral hippocampal RNS (11). Our observed median reduction in seizure frequency (26%) and responder rate (37.5%) are consistent with the 6–8 month outcomes in the RNS pivotal trial (12) and remain reassuring given observations that seizure reduction and response rates improve significantly over time with neurostimulation (13).

While most RNS outcomes are reported based on seizure diaries, we report results based on electrocorticographic “long events” that were detected and recorded automatically by the RNS device and then manually reviewed for accuracy. Long events were used for analysis because seizure diaries were not available for a few of the patients included in this study due to the short follow-up period. There is a strong correlation between reduction in patient-reported events and automatically recorded long events, however the relationship is not one-to-one (14). Long events generally overestimate patient-reported events. Large discrepancies between long events and patient-reported events have been reported (15), which introduces a degree of uncertainty in our results. However, it remains reassuring that our seizure response rates are consistent with other reported short-term results.

This study has limitations. The number of patients included is low, limiting the generalizability of the results. Furthermore, since our initial depth electrodes were placed in the supine position, with later electrodes placed in the prone position, a learning curve such as found by Faraji et al. (3) could account for some of the improvement in radial target error. Additionally, despite manual confirmation, errors in image registration and frame-robot registration could affect our analysis. The post-operative follow-up duration is relatively short, and we will continue to monitor these patients.

## Conclusion

Robotic stereotaxy enables submillimeter radial target accuracy for implantation of occipital -approach hippocampal RNS depth electrodes. In our experience, accuracy is better when the patient is positioned prone compared to supine slouched semi-sitting positioning; However, more studies with larger cohorts of subjects are required before generalizing the findings reported in this study. In addition to its excellent accuracy, robotic placement of these depth leads improves ergonomic comfort, increases operative efficiency by allowing parallel bilateral operation, and reduces the chance for human error associated with frame coordinates.

## Data Availability Statement

The original contributions presented in the study are included in the article/supplementary materials, further inquiries can be directed to the corresponding author/s.

## Ethics Statement

The studies involving human participants were reviewed and approved by Baylor College of Medicine Institutional Review Board. Written informed consent for participation was not required for this study in accordance with the national legislation and the institutional requirements.

## Author Contributions

PK and SS: drafted manuscript, critically revised manuscript, data collection, data analysis, and conceptualization. NG, JT, MP, AK, and BS: data collection, data analysis, conceptualization, and critically revised manuscript. JG, AM, ZH, PV, JC, and VK: data collection and analysis, conceptualization and interpretation of data, and critically revised manuscript. All authors contributed to the article and approved the submitted version.

## Conflict of Interest

SS is a consultant for Boston Scientific, Abbott, and Zimmer Biomet. The remaining authors declare that the research was conducted in the absence of any commercial or financial relationships that could be construed as a potential conflict of interest.
